# Long-term time-lapse live imaging reveals extensive cell migration during annelid regeneration

**DOI:** 10.1186/s12861-016-0104-2

**Published:** 2016-03-23

**Authors:** Eduardo E. Zattara, Kate W. Turlington, Alexandra E. Bely

**Affiliations:** Department of Biology, University of Maryland, College Park, MD 20740 USA

**Keywords:** Annelid neoblast, Cell migration, Developmental dynamics, Growth rates, In-vivo studies, Regeneration, Time-lapse imaging

## Abstract

**Background:**

Time-lapse imaging has proven highly valuable for studying development, yielding data of much finer resolution than traditional “still-shot” studies and allowing direct examination of tissue and cell dynamics. A major challenge for time-lapse imaging of animals is keeping specimens immobile yet healthy for extended periods of time. Although this is often feasible for embryos, the difficulty of immobilizing typically motile juvenile and adult stages remains a persistent obstacle to time-lapse imaging of post-embryonic development.

**Results:**

Here we describe a new method for long-duration time-lapse imaging of adults of the small freshwater annelid *Pristina leidyi* and use this method to investigate its regenerative processes. Specimens are immobilized with tetrodotoxin, resulting in irreversible paralysis yet apparently normal regeneration, and mounted in agarose surrounded by culture water or halocarbon oil, to prevent dehydration but allowing gas exchange. Using this method, worms can be imaged continuously and at high spatial-temporal resolution for up to 5 days, spanning the entire regeneration process. We performed a fine-scale analysis of regeneration growth rate and characterized cell migration dynamics during early regeneration. Our studies reveal the migration of several putative cell types, including one strongly resembling published descriptions of annelid neoblasts, a cell type suggested to be migratory based on “still-shot” studies and long hypothesized to be linked to regenerative success in annelids.

**Conclusions:**

Combining neurotoxin-based paralysis, live mounting techniques and a starvation-tolerant study system has allowed us to obtain the most extensive high-resolution longitudinal recordings of full anterior and posterior regeneration in an invertebrate, and to detect and characterize several cell types undergoing extensive migration during this process. We expect the tetrodotoxin paralysis and time-lapse imaging methods presented here to be broadly useful in studying other animals and of particular value for studying post-embryonic development.

**Electronic supplementary material:**

The online version of this article (doi:10.1186/s12861-016-0104-2) contains supplementary material, which is available to authorized users.

## Background

Animal development is a complex process in which numerous cells proliferate, move and differentiate in exquisitely coordinated fashion across space and time. The dynamic processes of animal development have traditionally been studied by examining select time points along a developmental trajectory and making inferences about intermediate steps between these data “still shots”. Often, these still shots must be collected from fixed tissue, precluding longitudinal studies of the same individual and requiring large sample sizes to account for inter-individual variability in the developmental process under study. More recently, time-lapse imaging, in which developing specimens are kept stationary and imaged continuously at short intervals of time, has proven to be an invaluable tool for understanding complex cell and tissue dynamics. Indeed, time-lapse imaging of live specimens, often coupled with fluorescent labeling, has led to major advances in our understanding of the developmental dynamics of model systems such as mice [1–4], chicks [5, 6], zebrafishes [7–10], nematodes [11–13] and fruit-flies [14] and has also been used widely for studying embryonic development in non-model species [15–17].

While time-lapse studies of embryonic development, including movies of long duration, have become relatively common [4, 7, 8], there is a clear dearth of comparable studies of post-embryonic development (e.g., regeneration, asexual reproduction, adult growth). Although some recent studies have used time-lapse imaging to capture relatively short time periods of post-embryonic processes [18–21], we are aware of no long-term longitudinal studies on invertebrates, and only a single recent study in a vertebrate system [10], that capture the full duration of processes such as regeneration or asexual reproduction. Successful long-duration time-lapse imaging requires keeping an animal immobilized but alive and healthy for a significant period of time within an optically clear support [17, 20]. While early embryos are usually static, movement becomes a major problem for imaging at later embryonic stages, when ciliary or muscular structures develop, and is a persistent impediment to imaging of typically motile juvenile and adult stages.

Regeneration of lost structures is an important post-embryonic developmental process that occurs in diverse animal groups and that typically occurs over several days to weeks [22–27]. Although there is much interest in regeneration, we are not aware of any continuous time-lapse recordings capturing the complete anterior and posterior regeneration process for any animal. Time-lapse imaging of regeneration would provide important insights into the cell and tissue dynamics of this process, revealing cell migrations, tissue movements and growth at fine spatial and temporal scales.

Here we describe a novel protocol to achieve long-duration continuous time-lapse imaging of anterior and posterior regeneration in adult annelid worms. We focus on the water nymph worm *Pristina leidyi* Smith (Annelida: Clitellata: Naididae), a small freshwater oligochaete that is well suited to studies of post-embryonic development. Adults are small (~200 μm diameter; ~2–6 mm length) and transparent; they typically reproduce asexually by paratomic fission, providing abundant and genetically homogenous material for study; and they exhibit robust and rapid regeneration, being capable of regenerating amputated anterior or posterior ends in just 3–5 days [28]. To illustrate the power of high spatial-temporal time-lapse imaging achievable with this new technique, we analyze the growth rate of the regenerate over the entire course of regeneration and characterize the cell migration response during early anterior and posterior regeneration.

## Results and discussion

The difficulty of immobilizing typically active adult animals over extended periods of time has been a long-standing challenge for studying post-embryonic development, thus far precluding long-duration time-lapse imaging of processes such as regeneration and asexual reproduction. We have developed a set of protocols that overcome this challenge in naidid annelids, enabling us to perform low- and high-magnification time-lapse microphotography of adults undergoing head or tail regeneration. Using tetrodotoxin (TTX) as a non-lethal immobilizing agent and mounting techniques that prevent dehydration while allowing for adequate gas exchange, we are able to continuously image regenerating worms for up to 120 h (5 days) under both dissection and compound microscopes. The methods presented here are relatively simple and likely to be adaptable for studying post-embryonic development in other animals.

### Tetrodotoxin causes non-lethal immobilization of naidid annelids and other animals

Successful long-duration time-lapse imaging requires immobilizing specimens but with minimal impact on survival, development and physiological processes. We tested the efficacy of a number of procedures to achieve benign immobilization of the annelid *Pristina leidyi* (see Methods). Most of the procedures we tested either were lethal or their immobilizing effects wore off upon prolonged exposures. Immersion in ice-cold culture water, nicotine, or chloretone immobilizes worms for a short period of time (5–15 min) but animals either die or habituate to these treatments if maintained longer. Ivermectin, which targets invertebrate glutamate-gated chloride channels [29], is an effective paralyzing agent in the short term, but worms typically die after a few hours of exposure. Paralyzing or anesthetic toxins, such as D-tubocurarine and dibucaine, were found to either have no effect at low doses, or be lethal at higher doses, with no useful immobilization in between. Since the process of regeneration takes place over several days, none of these compounds or procedures was found to be suitable for immobilizing worms for long-duration imaging; a recent screen in earthworms for anesthetics that could be useful for magnetic resonance imaging reported similar results, with only alcohol compounds yielding satisfactory immobilization over a few hours [30].

**Fig. 1:**
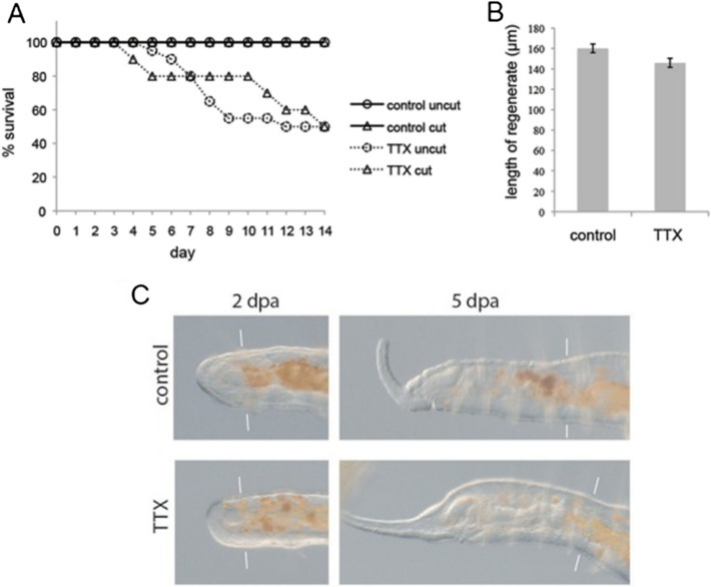
Effect of tetrodotoxin on survival and regeneration in *P. leidyi*. **a** Survival in TTX-treated and Control worms that were Cut or Uncut. **b** Regenerate length on Day 5 after anterior amputation in TTX-treated and Control worms. **c** Representative images of anteriorly regenerating worms that were TTX-treated or not treated, on days 2 and 5 after amputation (worms are from same experiment as panel **a**). Paired *white lines* indicate approximate amputation plane. Anterior is to the left in this and all figures

We ultimately found that one compound we tested, tetrodotoxin (TTX), produced the desired effects. TTX is known as a potent and selective blocker of sodium channels [31, 32]. In earthworms (also annelids), it acts as an irreversible blocker of skeletal muscle contraction [33] but fails to modulate gut muscle response to neurotransmitters [34]. We found that in *P. leidyi*, treatment with TTX (5–20 μM) for several hours effectively and irreversibly paralyzes worms. Importantly, TTX treatment does not inhibit in any obvious way the activity of smooth muscles of the gut and blood vessels, allowing apparently normal gut peristalsis and blood circulation. Normal functioning of digestive and circulatory systems in TTX-treated individuals likely synergizes with this species’ high tolerance to starvation (A. E. Bely and S. D. Brodsky, unpublished data), allowing for long-term survival of paralyzed individuals. Indeed, we found that either uncut or amputated individuals treated with TTX and then maintained in spring water have 100 % survival for the first 3 days of paralysis and exhibit only 50 % mortality even after 2 weeks (Fig. 1a). Importantly, we found that transferring animals to spring water after TTX incubation caused no decrease in paralysis; thus, TTX-treated animals can be removed from the TTX solution after drug incubation, making for much safer subsequent handling. Incubation at lower TTX concentrations or for shorter periods of time sometimes resulted in incomplete paralysis; however, we never observed recovery of normal mobility and behavior even under these conditions.

To assess the potential application of TTX-paralysis beyond *P. leidyi*, we tested the effects of TTX incubation in additional annelids including five other species of water nymph worms (Naididae), the blackworm *Lumbriculus variegatus* (Müller) (Lumbriculidae), the ghostworm *Aeolosoma hemprichi* Ehrenberg (Aeolosomatidae) and the bloodworm *Glycera dibranchiata* Ehlers (Glyceridae) (Table 1). We found that TTX is effective in inducing non-lethal paralysis in all species with the exception of *Aeolosoma hemprichi*, a tiny species that retained partial mobility, possibly because it moves largely by ciliary gliding. Thus, TTX may be broadly useful as a non-lethal paralyzing agent in annelids.

**Table 1:** Species tested for tetrodotoxin sensitivity

Phylum	Class	Species	Exposure mode & concentration	Effect	Mortality over 24 hours	Survival time	Notes
Annelida	Clitellata	*Pristina leidyi*	Immersion ~5–20 μM	Total paralysis	None	>10 days	
Annelida	Clitellata	*Paranais litoralis*	Immersion ~5–20 μM	Total paralysis	Low	3–5 days	
Annelida	Clitellata	*Dero digitata*	Immersion ~5–20 μM	Total paralysis	None	>5 days	
Annelida	Clitellata	*Nais stolci*	Immersion ~5–20 μM	Total paralysis	None	>5 days	
Annelida	Clitellata	*Allonais paraguayensis*	Immersion ~5–20 μM	Total paralysis	None	>5 days	
Annelida	Clitellata	*Stylaria lacustris*	Immersion ~5–20 μM	Total paralysis	None	>5 days	
Annelida	Clitellata	*Lumbriculus variegatus*	Immersion ~5–20 μM	Total paralysis	None	>5 days	
Annelida	Polychaeta	*Aeolosoma hemprichi*	Immersion ~5–20 μM	Partial paralysis	None	>5 days	Worms retain ciliary motion
Annelida	Polychaeta	*Glycera dibranchiata*	Injection ~20 μL, 6 μM	Total paralysis	None	>4 days	
Platyhelmintha	Tricladida	*Schmidtea mediterranea*	Immersion, injection, up to 6 mM	None	Not tested	NA	Moves by cilliary gliding
Hexapoda	Insecta	*Papilio polytenes*	Injection ~20 μL, 6 μM	High paralysis	Low	>3 days	Retains slow contraction/distension movement
Tardigrada		*Hypsibius dujardini*	Immersion up to 1 mM	None	Not tested	NA	
Nemertea	Heteronemertea	*Ramphogordius sanguineus*	Immersion, up to 60 μM	Low-medium paralysis	None	>5 days	Worms retain ciliary motion and keep gliding slowly
Nemertea	Hoplonemertea	*Prostoma eilhardi*	Immersion, up to 60 μM	None	None	>5 days	Worms retain ciliary motion and keep gliding slowly
Mollusca	Cephalopoda	*Loligo* sp.	Immersion, up to 60 μM	Medium paralysis	Low	1–2 days	Tested on squid hatchlings only

To examine responses to TTX outside of annelids, we also tested representatives of Plathyhelminthes, Hexapoda, Tardigrada, Nemertea and Mollusca (Table 1). We found that the movements of most species tested were greatly reduced by TTX. As with *Aeolosoma hemprichi*, we found that organisms that move primarily by ciliary gliding were not completely paralyzed by TTX treatment. However, TTX caused effective paralysis in organisms that move by muscular action, suggesting that our technique is potentially useful in a wide range of animals.

### TTX-treated animals appear to regenerate normally

Besides achieving full paralysis with high survival, an effective method for immobilization must not significantly affect the process under study. To test whether TTX treatment has an effect on the regeneration process, we amputated anteriorly both TTX-treated worms (*n* = 12) and control (untreated) worms (*n* = 12) and monitored them daily for 5 days. All TTX-treated worms survived to Day 5 and regenerated complete anterior ends. We detected no difference between the regeneration processes of TTX-treated animals with those of non-paralyzed controls (Fig. 1c). The average length of the regenerate (new tissues) on Day 5 was slightly smaller in TTX-treated worms (146 ± 15 μm) than in control worms (160 ± 13 μm) (Fig. 1b), but the difference was not statistically significant (Mann–Whitney rank sum test’s W = 86, *p* = 0.09). In both vertebrates and invertebrates, including *P. leidyi*, the developing blastema is normally innervated by nerve extensions from the stump [28, 35]. Our finding that TTX-treated *P. leidyi* appear to regenerate normally suggests that Na^+^ channel-dependent nerve conduction may not be required during regeneration. Alternatively, *P. leidyi* may possess multiple types of sodium channels, as seen in the genomes of the annelid *Capitella* and the mollusc *Lottia* [36], and one or more of these may be TTX-insensitive.

### Continuous time-lapse imaging reveals growth dynamics during regeneration

Still-shot developmental studies are useful for identifying a sequence of landmark events but are usually inadequate for studying fine-scale dynamics, as these often become evident only by observing the same individual over time. We used time-lapse imaging of TTX-immobilized *P. leidyi* to collect movies of high spatio-temporal resolution suitable for investigating the fine-scale dynamics of regeneration.

**Fig. 2:**
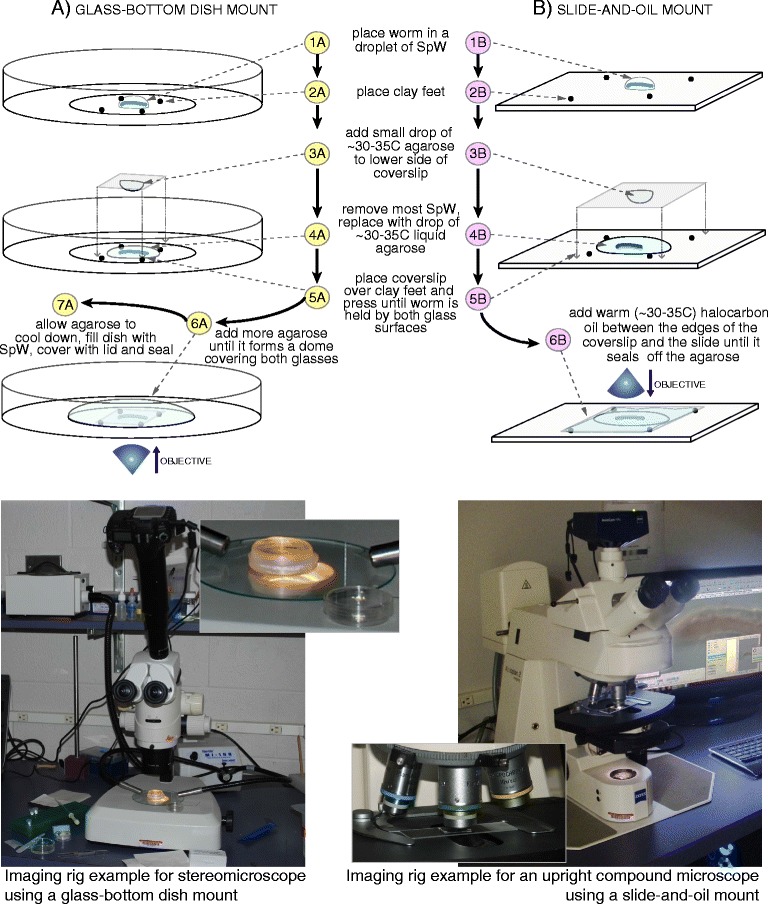
Workflow for tetrodotoxin treatment and sample mounting using the glass-bottom dish method (**a**) and the slide-and-oil method (**b**). Procedure steps are numbered and pictures at *bottom* illustrate the imaging setups used in the present study

We devised two methods of mounting TTX-paralyzed worms for time-lapse imaging, one using a glass-bottom dish, suitable for a large working distance setup such as a stereomicroscope, and the other using a glass slide, suitable for a small working distance setup such as a compound microscope (Fig. 2). For both mounting methods we first embedded specimens in agarose under a cover slip to further immobilize them. Then, to keep specimens from desiccating while allowing for sufficient gas exchange during the extended imaging period, for the dish mount we filled the dish with culture water and sealed it, while for the slide mount we surrounded the worm in agarose with halocarbon oil.

**Fig. 3:**
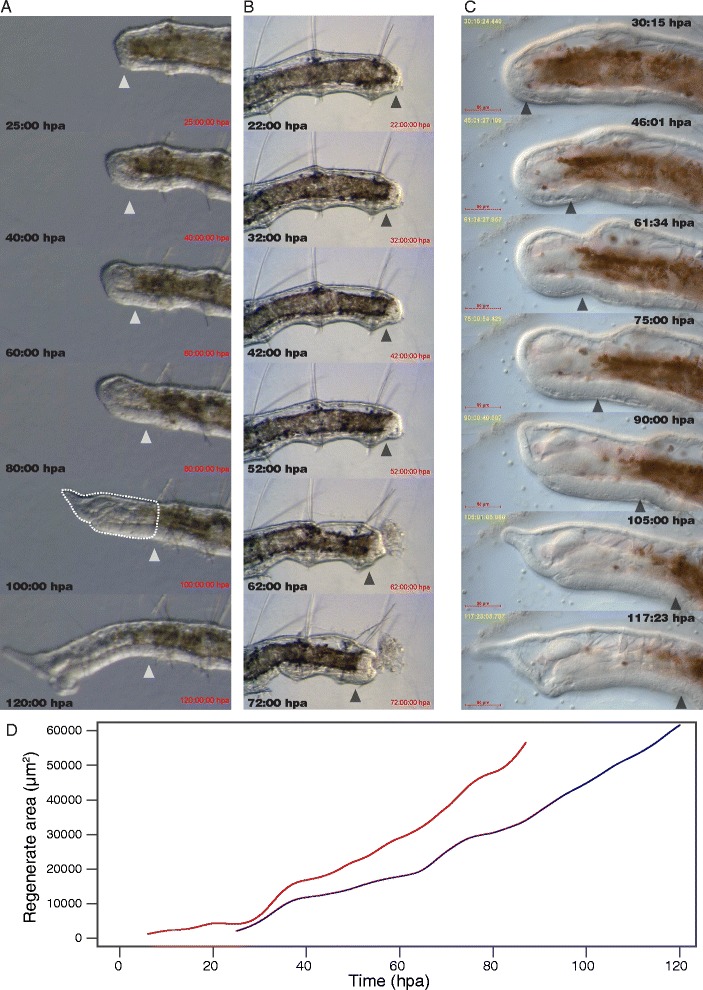
Time-lapse imaging and growth rate analysis during regeneration. **a** Frames from a time-lapse movie of anterior regeneration using a glass-bottom dish mount. Frames shown are 20 h apart, except for the first two frames shown which are 15 h apart. *Dotted outline* at 100 hpa illustrates how regenerate area was measured for growth rate analysis. **b** Frames from a time-lapse movie of posterior regeneration using a glass-bottom dish mount. Frames shown are 10 h apart. Defecation is evident in the 62 hpa frame, indicating the re-opening of the anus. **c** Frames from a time-lapse movie of anterior regeneration using a slide-and-oil mount. Frames shown are 15 h apart. In **a**-**c**, time is shown as hours post-amputation; amputation level is indicated by *arrowheads*. **d** Growth curves of anterior regenerate cross-sectional area over time for two different individuals (shown in red and purple).

Using these methods we are able to continuously image regenerating worms for up to 120 h (5 days), under either a dissection or compound microscope. This allowed us to record in amputated *P. leidyi* the entire process of anterior regeneration, including complete development of the prostomial/peristomial end, four chaetigerous segments and complete foregut and pharynx, and posterior regeneration, including complete development of the pygidium, posterior growth zone, a variable number of chaetigerous segments, and hindgut. Using the dish method, we acquired videos that capture at low magnification the processes of anterior regeneration of the head (Fig. 3a, Additional file 1: Movie 1) and posterior regeneration of the tail (Fig. 3b, Additional file 2: Movie 2) imaged every 5 min at a single focal plane. Using the slide-and-oil method, we were able to capture the complete process of head regeneration as a 4D dataset with high spatial and temporal resolution (Fig. 3c, Additional file 3: Movie 3; showing only a single focal plane). To our knowledge, this is the first report of complete anterior and posterior regeneration recorded at high temporal resolution and lasting several days (the longest reported recording of regeneration, tail fin regeneration in zebrafish, is 1.5 days long [10]).

Prior descriptions of *P. leidyi* regeneration have been based solely on still-shot observations at coarse time intervals, precluding a fine-resolution analysis of regeneration dynamics [28]. Using the techniques presented here, we were able to analyze hourly changes in the size of the regenerated tissues in recordings of complete anterior regeneration of two worms (Fig. 3, Additional file 1: Movie 1). We found that while the size of the developing head increases throughout the whole process, growth rates vary over time (Fig. 3d), with a very rapid acceleration starting about 30 h post-amputation (hpa), coincident with early blastema formation. 

Our time-lapse method also allowed us to image the effects of interactions between amputation and fission. A recent study has shown that *P. leidyi* individuals that are amputated and have active fission zones can respond in complex ways, including by accelerating fission at the expense of regeneration progression [37]. We imaged such an event, recording accelerated head development within the fission zone in an anteriorly amputated individual that failed to regenerate (Additional file 4: Movie 4).

### 4D imaging reveals widespread cell migration involving multiple cell types during regeneration

Although cell migration occurs widely during development, it typically occurs on a slow time scale making it difficult to observe directly. Cell migration has therefore often been inferred based on still-shot data, yet such data cannot provide direct evidence of migration and cannot reveal behaviors of individual migrating cells. Our time-lapse imaging methods were recently used to study the migration of individual cells during fission in *P. leidyi* [38], following an early description of our methods [39]. Here, we used high-resolution time-lapse imaging to record the post-amputation cell migration response and characterized the migration behavior of individual cells, providing the first analysis of post-amputation cell migration dynamics in annelids that we are aware of that tracks individual cells over time.

We collected 4D datasets from 6 uncut worms, 9 anteriorly cut worms, and 6 posteriorly cut worms (focusing on the 2–4 segments closest to the amputation site) and found that cell migration is common and widespread in both cut and uncut worms in *P. leidyi* (Additional files 5, 6, 7, 8, 9, 10 and 11: Movies 5–11). Most migrating cells were detected within the coelomic space, particularly over the parietal peritoneal lining and ventral nerve cord, but cells moving through other tissues (e.g., outer epidermis, gut lining) were also noted. Migration speeds for individual cells ranged from nearly 0 μm/min to over 30 μm/min. The detectability and traceability of cells varied considerably depending on the moving cell’s size, morphology and location. For example, cells moving in the coelomic cavity or crawling along the inner side of the body wall are often easy to detect and trace, while cells embedded within compact tissues or obscured by overlying structures (e.g., chaetae, chaetal sacs) are difficult or impossible to distinguish. Despite these detection biases, enough moving cells were detected to allow for a preliminary description of the characteristics of migrating cells. To further characterize migratory cells, we analyzed a subset of data consisting of 490 cell tracks recorded from 5 anterior amputees, 5 posterior amputees and 5 uncut worms, with cut animals analyzed during the first 24 h after amputation (see Methods).

**Fig. 4:**
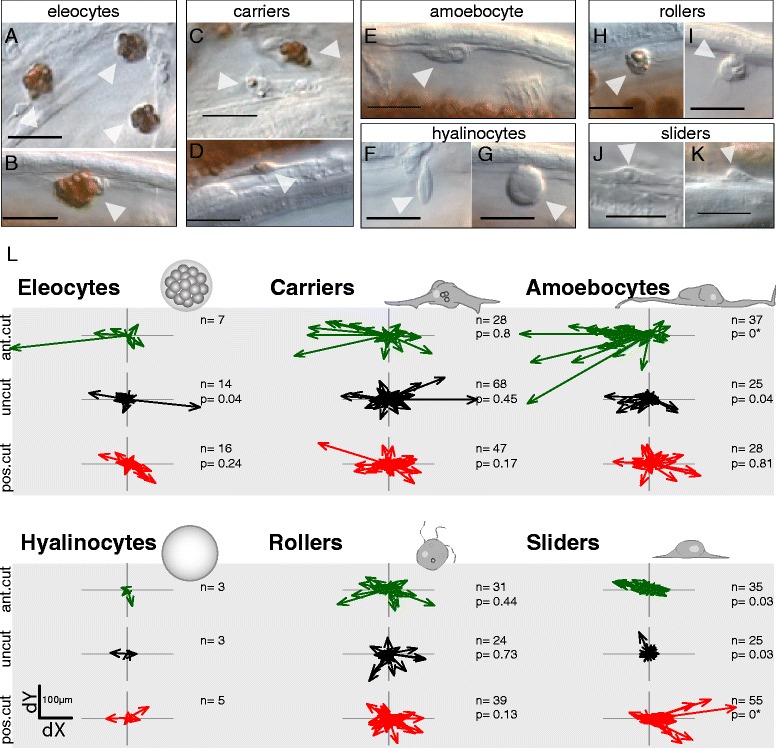
Six migratory cell populations recognized from analysis of 4D datasets. **a**-**k** Images of representative cells of each putative cell type (*white arrowheads*). *Scale bars*: 20 μm. **a** Round eleocytes clinging to a septum and chaetal muscles. **b** Migrating eleocyte sliding along the dorsal body wall. **c** Two carrier cells near the ventral surface of the coelom; note the variable morphology and presence of one to many granular inclusions. **d** A carrier moving along the ventral body wall. **e** An amebocyte moving along the dorsal body wall; these cells can be distinguished from amoeboid carriers by their larger size and different movement behavior. **f** A fusiform hyalinocyte, clinging by one end from the dorsal body wall. **g** A round hyalinocyte with one side attached to the dorsal body wall. **h** A roller with granular inclusions moving along the dorsal body wall. **i** A roller without inclusions moving along the dorsal body wall; note the fine filopodia. **j** A slider moving anteriorly along the peritoneal lining of the lateral body wall. **k** A slider moving posteriorly along the dorsal surface of the ventral nerve cord. **l** Vector plots of overall XY plane displacement of individual cells in anterior amputees (*green*), uncut worms (*black*), and posterior amputees (*red*). Each *arrow* represents the difference between XY coordinates measured at the start and end of the track of one cell (i.e., *longer arrows* represent larger total displacements); vectors are not corrected for track duration. Scale for all plots is shown at *lower left*. Shown at *right* for each vector group are the sample size (*n*) and the *p*-value of Wilcoxon rank sum tests for the average X displacement being significantly different from zero (calculated only for groups where *n* > 10; *p* = 0* indicates *p* < 0.001)

Based on the morphologies, locations, and movement behaviors of cells in this dataset, we categorized moving cells into six putative cell types (Table 2): eleocytes (Fig. 4a and b), “carriers” (Fig. 4c and d), amoebocytes (Fig. 4e), “hyalinocytes” (Fig. 4f-g), “rollers” (Fig. 4h-i) and “sliders” (Fig. 4j-k). For categories of cells that appeared to closely match previously described annelid cell types, we used these previously coined names [40–42]; where we had insufficient confidence to make such assignments, we have coined new names, placed in quotes above, as temporary placeholders until further evidence of their nature and homologies becomes available. We refer to these six categories as “cell types” in the remainder of this paper. However, we recognize that our cell type categories could very well include heterogeneous populations of similar cells and that these assignments are necessarily preliminary. Cell type categories will need to be further validated, ideally using molecular markers as these become available.

**Table 2:** Characteristics of migratory cell types. See Methods for abbreviations in table

Cell type	Characteristics	*N*	Cell size (μm)	Speed (μm/min)	Total mean square displacement (μm^2^)	Tangential trajectory speed ($\nu$) ALL / $\nu$ < 100	Timescale of autocorrelation ($\tau$) ALL/$\tau$ > 0.01
Eleocyte (Fig. 4a and b)	• Large, round, with a globular cluster of granules. • Found within coelomic cavity, single or in clusters attached to septa, gut, or chaetal sac muscles. • Can “jump” after detaching from anchor points, or crawl along the peritoneal lining.	37	avg: 12.7	avg: 3.5	avg: 6882	min: 1.30 / 1.30	min: 0.00 / 0.05
			rng: 9–19	med: 2.8	med: 1545	1stQ: 3.97 / 2.55	1stQ: 0.00 / 0.33
				max: 33.9	max: 122,671	med: 6.99 / 4.63	med: 0.37 / 0.84
						avg: 1716 / 5.50	avg: 2578 / 1.08
						3rdQ: 951 / 6.99	3rdQ: 1.27 / 1.41
						max: 16,210 / 18.64	max: 1.02e5 / 4.98
						n: 37 / 25	n: 37 / 25
“Carrier” (Fig. 4c and d)	• Medium size, variable irregular shape containing few to several granules. • Found within coelom. • Move by sliding and pseudopodial “walking”.	143	avg: 10.8	avg: 3.3	avg: 5167	min: 0.76 / 0.76	min: 0.00 / 0.03
			rng: 7–18	med: 2.5	med: 871	1stQ: 3.75 / 3.04	1stQ: 1e-5 / 0.42
				max: 20.1	max: 79,334	med: 6.91 / 4.86	med: 0.50 / 0.79
						avg: 801 / 5.60	avg: 1.00 / 1.42
						3rdQ: 568 / 7.10	3rdQ: 1.05 / 1.25
						max: 14,730 / 48.74	max: 32.8 / 32.8
						n: 143 / 101	n: 143 / 101
Amebocyte (Fig. 4e)	• Large amoeboid cells containing usually 1–2 large granules, sometimes more or none. • Found along the inner side of the body wall • Crawl mostly on dorsal peritoneum by extending long pseudopodia, stretching as much as 50 μm or more.	90	avg: 17.0	avg: 2.9	avg: 12,007	min: 1.06 / 1.06	min: 0.00 / 0.14
			rng: 8–30	med: 1.9	med: 3019	1stQ: 2.03 / 1.92	1stQ: 0.48 / 0.59
				max: 33.0	max: 170,254	med: 3.66 / 2.81	med: 0.97 / 1.03
						avg: 341 / 3.97	avg: 1.17 / 1.37
						3rdQ: 7.16 / 4.92	3rdQ: 1.51 / 1.56
						max: 9673 / 22.06	max: 6.35 / 6.35
						n: 90 / 77	n: 90 / 77
Hyalinocyte (Fig. 4f and g)	• Medium to large, round or oblong with smooth texture; no granules. • Typically found along the inner side of the body wall • No obvious motility structures, swing from an attached edge.	12	avg: 10.7	avg: 4.7	avg: 1518	min: 0.89 / 0.89	min: 8e-6 / 0.02
			rng: 8–16	med: 2.7	med: 601	1stQ: 3.27 / 3.01	1stQ: 0.06 / 0.92
				max: 11.3	max: 4586	med: 6.20 / 5.87	med: 1.06 / 1.18
						avg: 113 / 6.25	avg: 1.11 / 1.34
						3rdQ: 11.20 / 7.23	3rdQ: 1.64 / 1.70
						max: 743 / 17.93	max: 3.73 / 3.73
						n: 12 / 10	n: 12 / 10
“Roller” (Fig. 4h and i)	• Medium size, round cells, with none, one or two granules. • Found on coelomic cavity, in tangential contact with their substrate or detached. • Move by rolling over the substrate aided by small filopodia.	94	avg: 8.6	avg: 4.9	avg: 3047	min: 0.87 / 0.87	min: 0.00 / 0.07
			rng: 7–12	med: 4.7	med: 1555	1stQ: 5.66 / 5.23	1stQ: 0.28 / 0.57
				max: 21.3	max: 24,365	med: 6.85 / 6.47	med: 0.71 / 0.92
						avg: 1032 / 6.71	avg: 1.07 / 1.29
						3rdQ: 9.42 / 7.45	3rdQ: 1.36 / 1.43
						max: 26,140 / 21.27	max: 6.81 / 6.81
						n: 94 / 78	n: 94 / 78
“Slider” (Fig. 4j and k)	• Small to medium, spindle shaped cells, with no granules (rarely one). • Large surface in contact with the substrate, often over ventral nerve cord or lateral peritoneum. • Move by sliding over the substrate.	115	avg: 9.6	avg: 2.0	avg: 3071	min: 0.56 / 0.55	min: 0.00 / 0.04
			rng: 6–16	med: 1.2	med: 887	1stQ: 1.36 / 1.28	1stQ: 0.09 / 0.75
				max: 32.3	max: 66,262	med: 2.70 / 1.91	med: 0.86 / 1.22
						avg: 432 / 2.57	avg: 1.38 / 1.79
						3rdQ: 6.33 / 3.61	3rdQ: 2.17 / 2.48
						max: 11,030 / 8.95	max: 6.98 / 6.98
						n: 115 / 89	n: 115 / 89

### Movement patterns of individual cells show highly variable overall displacement, speed and directional memory

**Fig. 5:**
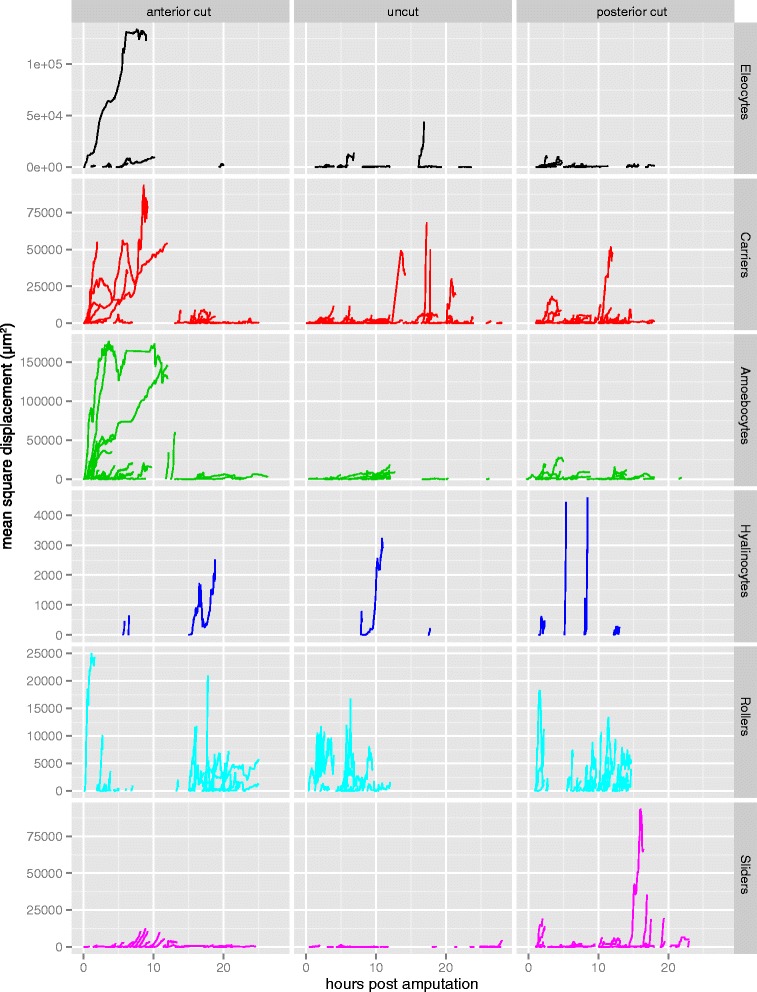
Mean square displacement of individual cell trajectories across time (hours post amputation; hpa), for all three treatments (columns) and six cell types (rows). Overall displacement of migrating cells is highly variable within and between cell types, treatments, and developmental time. Note that the vertical axis scale varies between rows

We then analyzed the movement patterns of individual cells during the first 24 h post amputation (hpa). Mean square displacement (msd) over time varied greatly between cells, both within and among treatments and cell types (Table 2; Additional files 5, 6 and 7: Movies 5–7; Fig. 5). Many cells have a large total msd, but many others show a much smaller displacement from their point of origin (Fig. 5). Strongly directional movement is detected in cells of several types and appears to be particularly common in anterior amputees during the first 10 h (e.g., steep upward curves in Fig. 5). Sliders, whose movements are detected throughout most of the time lapses, show directional movement during both anterior and posterior regeneration but most such movement occurs slightly earlier in anterior amputees (between 5 and 15 hpa in anterior amputees; between 10 and 20 hpa in posterior amputees). Although our cell scoring method does not ensure evenly distributed temporal sampling, a qualitative assessment of cell movement data suggests that anterior and posterior amputation contexts may affect cell behavior differentially.

**Fig. 6:**
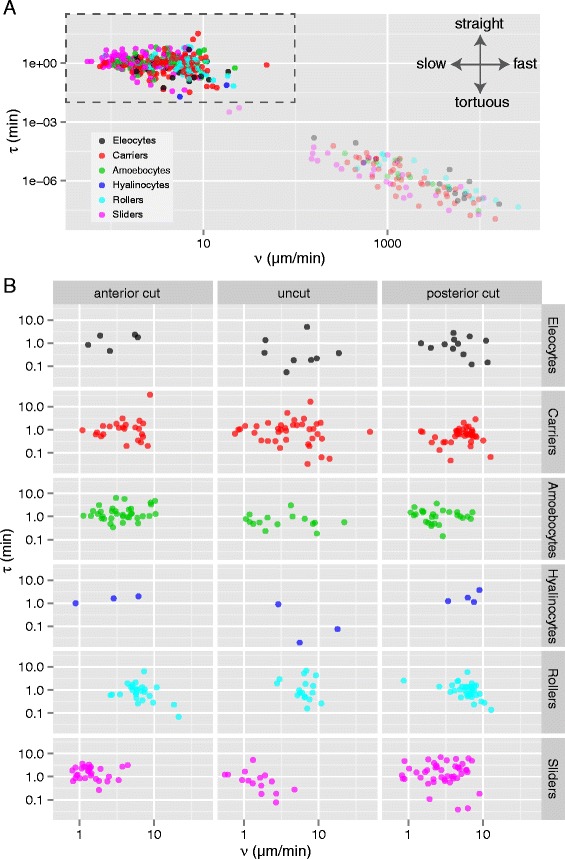
Speed and directional memory of individual cell trajectories. Most cell types show a similar range of speeds and directional memory. **a** Scatterplot of the timescale of autocorrelation (τ, in min) versus mean tangential speed (ν, in μm/min) for all cell types. Note that both axes are shown in log_10_ scale. **b** Scatterplots of τ versus ν for individual treatments (rows) and cell types (columns) for all cells within the boxed area in **a**

To further characterize the behavior of each cell, we used tools developed to analyze animal movement [43] to estimate two independent movement parameters: ν, the mean tangential speed of the trajectory, and τ, the time scale of autocorrelation (a measurement of the directional memory of the trajectory) (Table 2). Despite ν and τ being mathematically independent, plotting the estimates for these two parameters for all cells shows a clear negative correlation (Fig. 6). Cells cluster in two groups, one with high ν and low τ, and the other with low ν and high τ. Cells in the first cluster present trajectories with very short memory and thus very tortuous paths; their unrealistically high speed estimates are very likely the result of underestimation of τ (E. Gurarie, pers. comm.). Focusing specifically on cells in the second cluster (boxed region in Fig. 6a), we find that the parameter distribution is not obviously structured by cell type or treatment (Fig. 6b), except perhaps for sliders, which tend to show faster speeds in posterior amputees than in anterior amputees or uncut worms. Thus, it is possible that the same general range of movement behaviors is available to most moving cells, independent of cell type.

### Some cell types display strongly directional movement and preferential migration routes

We calculated migration speeds for individual cells and found that different types of migratory cells had similar average and median migration speeds but differed in overall movement trends. Average and median migration speeds ranged from ~2–5 μm/min across the different cell types, with individual cells exhibiting migration speeds of as little as nearly 0 μm/min to top recorded speeds of 11–33 μm/min (Table 2). Total displacement, however, varied within and between cell type and treatment (Fig. 4l). We analyzed whether cells moved preferentially in an anterior or a posterior direction and found that in many cases there was no statistically significant preference between anterior movement and posterior movement (Wilcoxon rank sum tests of difference from zero overall X-axis directionality; test performed only for groups with >10 cells scored). However, we found that predominantly anterior movement was significant for eleocytes in uncut worms (*p* = 0.04; *n* = 14), for amoebocytes in uncut worms (*p* = 0.04; *n* = 25) and anterior amputees (*p* = 9E-7; *n* = 37), and for sliders in uncut worms (*p* = 0.03; *n* = 25) and anterior amputees (*p* = 0.03; *n* = 35). In contrast, predominantly posterior movement was only significant for sliders in posterior amputees (*p* = 9E-7; *n* = 55). Interestingly, while anterior bias is statistically significant in uncut animals for amoebocytes and sliders, the mean absolute X-axis displacement is still significantly lower than in anterior amputees (amoebocytes: 37.7 versus 114.5 μm, Wilcoxon rank sum test *p* = 1.6E-5; sliders: 11.2 versus 36.7 μm, Wilcoxon rank sum test *p* = 1.4E-5). Thus, even if directionality is detected in the absence of injury, the magnitude of displacements towards the wound site is considerably greater in injured animals.

**Fig. 7:**
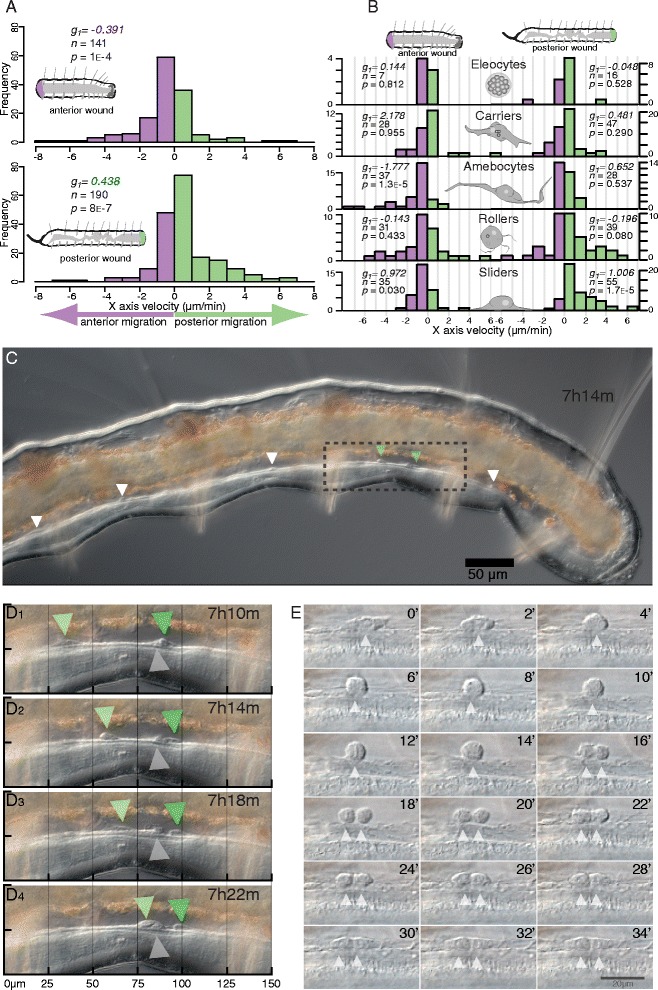
Directional migration of cell types during regeneration. **a**-**b** Histograms of cell X-axis velocities, measured as overall X displacement of a track divided by the track total duration, for cells tracked in 5 anterior (*top*) and 5 posterior (*bottom*) amputees. *Purple bars* represent anterior migration (negative values); *green bars* represent posterior migration (positive values). g1: skewness; n: number of cell tracks; p: *p*-value for the Wilcoxon rank sum test. Plots are made for anterior amputees and posterior amputees for all cells combined (**a**) and for each cell type (**b**); hyalinocytes were not included due to low sample size. **c** Sample XY slice from a 4D dataset of a posterior amputee (mid-sagital plane, approximately 7.5 hpa). *Arrowheads* indicate migrating sliders; *green arrowheads* highlight sliders shown in d (*boxed area*). **d** Detail of four XY slices of the *boxed region* in c spaced 4 min apart. Two ventral sliders (*green arrowheads*) are moving posteriorly at different speeds (*light green*: faster, *dark green*: slower). A third cell of about the same size as the sliders (*grey arrowhead*) remains in the same location. **e** Detail of a 4D dataset of an anterior amputee showing mitotic activity of a migrating cell. Shown are 18 slices, each spaced 2 min apart. While moving in an anterior direction over the peritoneal lining of the lateral body wall, a slider stops, rounds-up, and divides; the daughter cells then regain the spindle shape. Notice the transient vertical structure at 12′, presumably a metaphase plate

We then analyzed the velocity of cells along the anterior-posterior axis. Since specimens were all imaged in lateral view with anterior to the left, we used displacement along the X-axis in these datasets to estimate antero-posterior (AP) movement. Because worms tend to curl ventrally slightly, this approach may slightly underestimate actual AP displacement; using a customized reference axis for each dataset would be needed to correct for this, but we favored a simpler approach for this initial analysis. Since the durations of cell tracks ranged considerably, from less than 10 min to several hours depending on how easy it was to follow a particular cell, we divided total X-displacement by track duration for each cell to calculate X-axis velocity. We first investigated the behavior of all moving cells, independent of cell type. Histograms of X-axis velocity for individual cells across 5 anterior (141 cell tracks) and 5 posterior (190 cell tracks) amputees show negative skewness (*g*_*1*_ = −0.391) for anterior amputees and positive skewness (*g*_*1*_ = 0.438) for posterior amputees (Fig. 7a). The center of the distribution of X-velocities is significantly less than zero for anterior amputees (Wilcoxon rank sum test, *p* = 0.0004), while it is significantly greater than zero for posterior amputees (Wilcoxon rank sum test, *p* = 7E-7). Thus, following either anterior or posterior amputations, cell movements occur predominantly towards the wound site.

To determine whether this migration trend was common to all cell types, the same analysis was stratified by cell type (Fig. 7b). We found that the center of the distribution was only significantly different from zero for amoebocytes in anterior amputees (Wilcoxon rank sum test, *p* = 1.3E-5) and for sliders in both anterior and posterior amputees (Wilcoxon rank sum tests, *p* = 0.03 and *p* = 1.7E-5, respectively). Among uncut worms (159 cell tracks), only amoebocytes tended to move directionally, with a preference for anterior migration (Wilcoxon rank sum test, *p* = 0.009).

Regarding where in the body migratory cells were detected, we found that this location could differ between our treatments. Amoebocytes were detected in a similar location in uncut worms and amputees, being almost always found crawling along the dorsal surface of the coelomic cavity. However, sliders migrating in posterior amputees were most often found moving along the dorsal surface of the ventral nerve cord (47/55 cell tracks, Fig. 7c-d, Additional file 8: Movie 8 and Additional file 9: Movie 9) while sliders migrating in anterior amputees were most often found moving over the peritoneal lining of the lateral body wall surface (34/35 cell tracks, Additional file 10: Movie 10). Imaging of sliders moving along the lateral body is often particularly clear and, in two instances, we captured an anteriorly-moving slider stopping, rounding up from a spindle shape to a spherical shape, and undergoing a round of cell division; daughter cells then regained the typical slider shape and continued migrating (Fig. 7e, Additional file 11: Movie 11). Capture of only two mitotic events from 9 worms suggests cell division is infrequent during the first day after amputation, a finding that is consistent with the post-amputation mitotic shutdown previously described in this species [37]. In summary, our time-lapse imaging studies indicate that one putative cell type, which we call sliders, migrates to both anterior and posterior wound sites, appears to move along predominantly different routes during anterior and posterior regeneration, and at least during anterior migration can stop and divide while migrating towards the wound site.

### Directional migration of ventral sliders could be the first direct evidence of annelid neoblast migration

The prevailing model of regeneration in clitellate annelids postulates that after amputation a population of cells called annelid neoblasts migrates to the wound site and participates in the regeneration process [42, 44–47]. Many authors have in addition suggested that neoblasts are reserve stem cells, migrating to the wound site to provide a source of cells that can rebuild certain lost tissues, though others have disputed this claim [44, 47–58]. Support for neoblast migration comes from numerous still-shot studies, but to our knowledge such migration has never been demonstrated directly nor have the behaviors of individual neoblast cells ever been characterized.

The slider cells we identified in our time-lapse movies closely match descriptions of neoblasts made from static images of a range of clitellate annelid species. Neoblasts have been reported to be spindle-shaped cells, with a large nucleus to cytoplasm ratio, located primarily on the dorsal surface of the ventral nerve cord, and, based on sequential still shots during regeneration, inferred to migrate to wound sites [42, 46, 51, 56, 59]. The slider cells of *P. leidyi* match this description, strongly suggesting that these cells in whole or in part comprise the same cell type that has been referred to as neoblasts in other clitellate species. Assuming that this is indeed the case, to our knowledge, our time-lapse data represent the first direct evidence of migration of neoblast cells, originally proposed over 120 years ago [45, 46].

A long-standing question in annelid regeneration biology is what distinguishes the regenerative processes of anterior vs. posterior regeneration. Posterior, but not anterior, migration of neoblasts along the nerve cord has been described for *Tubifex*, *Limnodrilus* and *Allonais* (Naididae); in contrast, migration to both anterior and posterior wounds along the ventral nerve cord has been reported in *Dero* (Naididae) and *Lumbriculus* (Lumbriculidae) [49, 51, 53–55, 57–60]. In *P. leidyi*, we found morphologically similar slider cells moving towards both anterior and posterior wounds, but along different primary migration routes; sliders moving towards anterior wounds do so primarily along the lateral body wall, while sliders moving towards posterior wounds do so primarily over the ventral nerve cord. Whether anterior and posterior wounds recruit different cell types, and whether anteriorly migrating and posteriorly migrating sliders are the same cell type, several cell types, or even each heterogeneous populations of cells, remain important questions that must await future studies and the development of molecular markers for these cells.

Our time-lapse data also provide novel insights into the individual behaviors of sliders, which we posit to be putative neoblasts. Based on still-shot data, neoblasts have been inferred to migrate towards wounds, and based on this inference there has been substantial speculation about the role of neoblasts at the wound site, much of it focused on these cells being a possible source of regenerated tissues [42, 44, 50]. Our time-lapse data indicate, however, that although sliders move predominantly toward wound sites, some instead move *away* from wound sites. There has not been any proposed function for neoblasts that move away from wounds because such movement has never been inferred from still-shot data, highlighting the value of time-lapse imaging to reveal the behavior of individual cells. The unexpected dual migration direction of sliders should prompt the formulation of new hypotheses for the function of these cells, as well as further studies to determine whether what appears to be one cell population is in reality a heterogeneous cell population. While the final destinations of slider cells at the wound site could not be determined from our movies, coupling techniques to label these cells with time-lapse imaging could be a powerful approach to investigate the long-standing question of the fate of annelid neoblasts at wound sites.

## Conclusions

Here, we report a new technique for prolonged time-lapse live imaging of annelid regeneration. Using tetrodotoxin to immobilize specimens and mounting methods that prevent desiccation and allow for long-term survival, we have been able to generate high-resolution longitudinal recordings of the complete process of anterior and posterior regeneration. To our knowledge, these represent the most extensive recordings of regeneration in an invertebrate. These time-lapse recordings provide detailed information about the growth dynamics of regeneration and reveal extensive cell migration of several cell types following amputation. One of the migratory cell types, which we termed "sliders", matches the description of annelid neoblasts, putative stem cells long thought to be fundamental for regeneration success in annelids. We expect the methods described here will lead to important further insights into the cellular dynamics of regeneration and can be broadly useful in the study of animal post-embryonic development.

## Methods

### Animal culture

*Pristina leidyi* were maintained in bowls of artificial spring water (SpW, 1 % artificial seawater) and fed dried *Spirulina*.

### Tetrodotoxin-mediated immobilization

A range of methods/compounds were tested for immobilization efficacy and effect on survival in *P. leidyi*: ice-cold water (up to 15 min), nicotine (0.05–1 mM), chloretone (0.1 %), ivermectin (0.5–1 mM), D-tubocurarine (1–100 μM), dibucaine (0.1–10 mM) and tetrodotoxin (TTX) (5–20 μM). Because TTX provided the greatest advantages for immobilization, all remaining experiments used this method of immobilization.

Long term immobilization of *P. leidyi* was achieved by incubation with tetrodotoxin. Batches of 10–50 worms were transferred from cultures into 1.5 mL microcentrifuge tubes, washed several times with SpW to remove culture debris, and placed in 500 μL SpW. 0.5–3 μL of a 6.3 mM TTX stock solution (Sigma-Aldrich #T5651) was then added to the worms, yielding a final concentration of ~5–20 μM TTX. After gently mixing, worms were incubated in this solution at room temperature for 4–16 h, until worms stopped visibly moving. Since TTX is a highly potent and essentially irreversible toxin, after TTX incubation worms were washed three times with and subsequently maintained in SpW, allowing for safer subsequent handling of immobilized animals. Used TTX working solution and washes were stored and disposed of following UMCP’s Department of Environmental Safety guidelines. Stock solutions of TTX tend to lose potency after several months stored at 4 °C; higher concentrations of older stocks were used to compensate for this.

Besides *P. leidyi*, we tested the effect of TTX treatment in eight annelids, including *Allonais paraguayensis*, *Dero digitata*, *Nais stolci*, *Paranais litoralis*, and *Stylaria lacustris* (see Zattara & Bely [61] for culture sources); *Lumbriculus variegatus* (Carolina Biological Supply); *Aeolosoma* sp. (Carolina Biological Supply); and *Glycera dibranchiata* (Marine Biological Laboratory at Woods Hole, Marine Resource Department). In addition, we tested the planarian *Schmidtea mediterranea* (provided by A. Sánchez-Alvarado), the larval stage of the swallowtail butterfly *Papilio polytes* (provided by N. Patel), the water bear *Hypsibius dujardini* (Carolina Biological Supply), the marine ribbon worm *Ramphogordius sanguineus* (collected from Nahant, MA by J. Norenburg), the freshwater ribbon worm *Prostoma eilhardi* (collected from Concord, Eastbrook Woods, MA by C. Laumer), and the squid *Loligo* sp. (Marine Biological Laboratory at Woods Hole, Marine Resource Department). Individuals (*n* > 5) were exposed to TTX by immersion in ~5–20 μM TTX except for *Glycera*, *Schmidtea*, and *Papilio* which were injected with TTX using pulled glass needles. Individuals were scored for mobility within the first 24 h after treatment and survival was scored for 1–10 days depending on species.

### Amputations

To elicit regeneration in *P. leidyi*, worms were immobilized with TTX and amputated with a scalpel. For anterior amputations, the anterior 4–7 segments were removed. For posterior amputation, worms were cut after segment 14 and the posterior portion discarded.

### Effect of tetrodotoxin on survival and regeneration

We first tested the effect of TTX on survival and regeneration. Sixty *P. leidyi* worms were isolated from a culture and divided into four treatments: Control & Uncut (*n* = 20), Control & Cut (*n* = 10), TTX-treated & Uncut (*n* = 20), and TTX-treated & Cut (*n* = 10). TTX-treated worms were treated with TTX as described above and then moved to SpW after paralysis; Control worms were kept in SpW; Cut worms were amputated anteriorly, removing the anterior-most four segments; Uncut worms were left unamputated. Worms were maintained individually in ~ 2 ml SpW in wells of 24-well tissue culture plates. Worms were scored daily for survival and regeneration progress for 14 days and imaged on days 2 and 5 of the experiment, with Control worms relaxed with 0.05 mM nicotine for imaging.

We then tested the effect of TTX on regeneration and regenerate length. This experiment was similar to the first except that there were only two treatments, Control & Cut (*n* = 12) and TTX-treated & Cut (*n* = 12); worms were amputated anteriorly removing the anterior-most 4 segments; worms were scored daily from 0 to 5 days after amputation; and on day 5 all worms were fixed with 4 % formaldehyde in phosphate buffer saline and imaged under a calibrated compound microscope (see below) and the length of the anteriorly regenerated tissue was measured.

### Glass-bottom dish mounting method

For imaging with a large working distance or inverted microscope, we developed a mounting method using a glass-bottom dish (Fig. 2, left column). A TTX-immobilized worm was placed in a drop of SpW (~10 μL) on a glass bottom dish (P35G-0.17-14-C, MatTek, Ashland, MA) and four small balls of clay were placed around the drop. A small drop of warm (~30–35 °C) liquid low-melt agarose (SeaPlaque™ Agarose, #50101, Lonza, Basel) was then placed on the lower surface of a square piece of coverslip glass (~9 × 9 mm), using a glass pipette pre-warmed by pipetting warm agarose several times. Immediately, the SpW around the worm was replaced with ~20 μL of warm agarose and the coverslip glass with its agarose drop facing down was quickly placed on top of the worm over the clay balls. The coverslip was pressed down evenly until the worm was slightly pressed between the coverslip and the glass bottom of the dish. Warm agarose was then added first to the sides of the coverslip and then to the top of the coverslip, so that the circular cut-out portion of the dish bottom was covered by an agarose dome. Agarose was allowed to solidify and the dish was filled with SpW. The dish was then sealed by placing the lid of the dish on top and sealing the edges with Parafilm. We found this mounting method well suited to imaging with either an upright stereomicroscope (setting up the dish bottom up) or an inverted compound microscope (setting up the dish bottom down). Mounts made with this method are easier to set up and there is no desiccation risk, but due to the thickness of the glass and the plastic surface of the lid, it is harder to achieve the illumination necessary for enhanced contrast microscopy (e.g. DIC/Nomarski) using transmitted light.

### Slide-and-oil mounting method

For imaging with a small working distance microscope, we developed a slightly different mounting method using a slide and an oil seal (Fig. 2, right column). A TTX-immobilized worm was placed in a drop of SpW on a glass slide previously coated with Rain-X (ITW Global Brands, Houston, TX). Largely similar to the description above, four balls of clay were placed around the drop; a drop of warm agarose was placed on the lower surface of a #1.5 coverslip glass; the SpW around the worm was replaced with ~20 μL of warm agarose; and the coverslip glass with its agarose drop facing down was quickly placed on top of the worm over the clay balls. The coverslip was then pressed down evenly until the worm was slightly pressed between the coverslip and the slide surface. The mount was sealed using halocarbon oil (#FLY-7000, LabScientific, Livingston, NJ), which prevents water evaporation while allowing for gas exchange. Oil was applied all around the edges of the coverslip so that oil would seep between the coverslip and the slide, encircling the worm in agarose with oil. Warming the oil to 30–35 °C reduces viscosity and facilitates the capillary movement of the oil between the slide and coverslip. Obtaining good mounts with this method requires more practice than with the above glass-bottom dish method, but it allows for imaging with compound scopes with smaller working distances and specialized illumination techniques (e.g., DIC/Nomarski). Given the high refractive index of both water and agarose, using water immersion objective lenses with higher numerical aperture and longer working distance can greatly improve imaging at higher resolutions.

### Imaging with stereomicroscopes

Worms mounted with the glass-bottom dish method were imaged with two alternative stereomicroscope set-ups: an Olympus SZ60 stereomicroscope (Olympus, Tokyo, Japan) equipped with a DinoEye AM423x eyepiece camera (BigC.com, Torrance, CA, USA) and a Leica MZ16 stereomicroscope (Leica, Wetzlar, Germany) equipped with a Nikon D80 DSLR camera (Nikon, Tokyo, Japan). Both cameras were interfaced to a PC computer through USB serial ports and operated using manufacturer’s software (Fig. 2, lower left). The microscope was focused at a focal plane of interest and images were captured at regular intervals (2–5 min) with a pixel resolution of 1024 × 768 (DinoEye) or 1936 × 1296 (Nikon D80). Images were then compiled as single XYT stacks, cropped and exported as uncompressed or JPG compressed AVI video files using ImageJ [62]. Video file post-processing and re-encoding, if needed, was done using VirtualDub (http://www.virtualdub.org) and online editing at YouTube (http://www.YouTube.com).

### Imaging with compound microscopes

To record high-resolution 4D (XYZT) time-lapse stacks, worms mounted with the slide-and-oil method were imaged with DIC optics on a Zeiss Axioplan2 microscope equipped with a Zeiss AxioCam HRc camera using the 6D Acquisition module of AxioVision v4.8.2 (Zeiss, Oberkochen, Germany). We used a custom-made stage micrometer to calibrate images, enabling us to derive absolute measurements from them. Z-stacks comprising focal planes spaced 3 μm apart were acquired every 2 min. Imaging captured the wound site and up to four adjacent segments. Movies of selected focal planes were generated using AxioVision.

### Analysis of growth rates during anterior regeneration

Regeneration growth rates were analyzed from two anterior regeneration datasets, one spanning 6 to 87 h post amputation (hpa) and another spanning 25 to 120 hpa (glass-bottom dish mount, stereomicroscope imaging). Using ImageJ we extracted subsets of frames 1 h apart and measured the cross-sectional area of the entire anterior regenerate (all new tissues). Both time series were fit to a polynomial smoothing spline (sm.spline, pspline R package) and plotted over time after amputation to obtain growth curves.

### 4D dataset cell tracing and analysis

We scored 4D datasets (slide-and-oil mounts, upright compound microscope imaging) for a total of 21 worms: 9 anterior amputees (cut around segment 5), 6 posterior amputees (cut around segment 14) and 6 non-amputated controls. Worms were oriented with anterior to the left and posterior to the right, so the amputation plane is at the left end in anterior amputees and to the right in posterior amputees. All worms were imaged starting at 0–4 h post-amputation (hpa) through to 6–84 hpa (average: 28 hpa), or comparable start and end times for uncut worms. Because the speed of regeneration can differ slightly between individuals, causing their developmental stages to diverge over time, we limited our analysis to the first 24 hpa for cut worms or the first 24 h for uncut worms to maximize the match in developmental stages across replicates. Moving cells were identified visually, then measured, described and tracked frame-by-frame by scoring their XYZT coordinates using ImageJ’s measuring tools (X and Y were measured in μm; Z was calculated based on slice position and the known 3 μm Z-stack interval; and T was calculated from position along the time series). Although the antero-posterior (AP) and dorso-ventral (DV) axes were approximately aligned with the X and Y axes respectively, worms tended to curl ventrally, resulting in a slight curvature. Thus, XY coordinates only approximate actual AP and DV positions.

Visual cell scoring resulted in 21,227 sets of XYZT coordinates corresponding to 626 individual cell “tracks”. These tracks were the units of analysis: each is composed of an XYZT series of variable length representing the position of a given cell at each time point during the interval we were able to reliably follow that particular cell. Within each track, “instantaneous” displacement and speed at each time point were calculated as the geometric distance in XYZ position between two consecutive time points (i.e. the displacement every 2 min). For each track, we also calculated overall mean speed (the average of instantaneous movements across the complete track), maximum instant speed (the maximum value of instantaneous movements across the complete track), total displacement (difference between the first and last time points of the track), and overall velocity (total displacement divided by the track duration).

A subset of 491 tracks from 5 anterior amputees, 5 posterior amputees and 5 uncut worms was further characterized by re-examining the cells in each track and measuring their size, cell shape, presence of locomotive structures, attachment mode to substrates, and presence and density of cytoplasmic granules. These characteristics were used to categorize the cell in each track into one of six putative cell types. Trajectories of moving cells were analyzed individually, first by calculating mean square displacement (msd) for each data point, and then by fitting each trajectory to continuous-time correlated random walk (CRW) and continuous-time correlated velocity (CVM) models [43] using a wrapper of the crawl R package [63] (E. Gurarie, unpublished). For each cell, we estimated ν, the mean tangential speed, and τ, the timescale of autocorrelation (a measure of the directional memory of the trajectory). We also calculated summary statistics for each cell type: average (avg), range (rng), median (med), minimum (min), maximum (max), first quartile (1stQ), third quartile (3rdQ) and sample size (n). Directional preferences along the antero-posterior axis were tested statistically as differences from zero in the center of displacement and velocity distributions using the non-parametric Wilcoxon rank sum test, with α = 0.05. Statistical analysis and plotting were made in R [64]; R code and data tables are available in the Zenodo repository [65].

### Availability of data and materials

The datasets supporting the conclusions of this article are available in the Zenodo repository (DOI: 10.5281/zenodo.45833; http://zenodo.org/record/45833) [65]. Movies also accessible at https://goo.gl/Xp5nWv.

## Additional files

Additional file 1: Movie 1. Anterior head regeneration in the annelid *Pristina leidyi.* Ninety-five hour recording of full anterior regeneration after head amputation, spanning 25 to 120 hpa. The worm was paralyzed with tetrodotoxin, mounted in a glass-bottom dish and imaged with a 5-min frequency using a Nikon D80 DSLR camera mounted on a Leica stereomicroscope. Alternative link: https://youtu.be/xnfdjFJyeFc. (MP4 2.31 mb) Additional file 2: Movie 2. Posterior regeneration in the annelid *Pristina leidyi*. Seventy-one hour recording of posterior regeneration after posterior end amputation, spanning 2 to 73 hpa. The worm was paralyzed with tetrodotoxin, mounted in a glass-bottom dish and imaged with a 5-min using a DinoEye eyepiece camera on an Olympus stereomicroscope. Alternative link: https://youtu.be/iEe4STdJNhs. (MP4 2.09 mb) Additional file 3: Movie 3. Anterior head regeneration in the annelid *Pristina leidyi* at high resolution. High-spatial, fine-temporal resolution time-lapse recording of the complete process of anterior head regeneration in the annelid *Pristina leidyi*. The specimen was mounted using the slide-and-oil method and imaged over a period of 120 h after amputation using a Zeiss Axioscope 2 compound microscope. Magnification is at 100× (0–35 s movie time; 0–30 hpa real time) and subsequently 200× (36+ seconds movie time; 30+ hpa real time). Complete Z-stacks at 3 μm intervals were captured every 2 min. This video shows a complete T-series from a single focal plane from the 4D dataset. Alternative link: https://youtu.be/tnx-gU66-P0. (MP4 35.5 mb) Additional file 4: Movie 4. Interaction between regeneration and paratomic fission in the annelid *Pristina leidyi*. Time-lapse recording of a paratomically fissioning individual that has been amputated in front of the fission zone. The worm was paralyzed with tetrodotoxin, mounted in a glass-bottom dish and imaged at 5-min intervals using a DinoEye eyepiece camera on an Olympus stereomicroscope. While a non-fissioning individual would typically initiate head regeneration at the wound site, the presence of a fission zone in this individual appears to stall the regeneration process. Instead, head development within the fission zone appears to be accelerated. Alternative link: https://youtu.be/ZMdnzvpmZbI. (MP4 3.58 mb) Additional file 5: Movie 5. Animated example of cell tracing in an anterior amputee. Frame-by-frame scatterplot of XY cell position, changing over time after amputation, for a worm that has been anteriorly amputated. Each moving cell is color coded by cell type, and every past position is retained in order to visualize cell trajectories in space. Time after amputation is shown over the plot, and average and standard deviation of instantaneous cell speed for all presently moving cells are shown at the bottom of the plot. Anterior is to the left. Alternative link: https://youtu.be/hSlERtrrypY. (MP4 8.19 mb) Additional file 6: Movie 6. Animated example of cell tracing in a non-amputated control worm. Frame-by-frame scatterplot of XY cell position, changing over time, for a control worm that has not been amputated. Each moving cell is color coded by cell type, and every past position is retained in order to visualize cell trajectories in space. Time since beginning of experiment is shown over the plot, and average and standard deviation of instantaneous cell speed for all presently moving cells are shown at the bottom of the plot. Anterior is to the left. Alternative link: https://youtu.be/BMwxLg5bbHU. (MP4 4.93 mb) Additional file 7: Movie 7. Animated example of cell tracing in a posterior amputee. Frame-by-frame scatterplot of XY cell position, changing over time after amputation, for a worm that has been posteriorly amputated. Each moving cell is color coded by cell type, and every past position is retained in order to visualize cell trajectories in space. Time after amputation is shown over the plot, and average and standard deviation of instantaneous cell speed for all presently moving cells are shown at the bottom of the plot. Anterior is to the left. Alternative link: https://youtu.be/zQAqgciKurw. (MP4 3.90 mb) Additional file 8: Movie 8. Neoblast-like cells migrating along the nerve cord of a regenerating annelid worm. High-spatial, fine-temporal resolution time-lapse recording of posterior regeneration after posterior fragment amputation. The specimen was mounted using the slide-and-oil method and imaged using a Zeiss Axioscope 2 compound microscope. This clip shows the movement of neoblast-like slider cells on the dorsal surface of the nerve cord in a posteriorly regenerating annelid, *Pristina leidyi*. Green arrowheads highlight cell movement towards the posterior of the animal, while magenta arrowheads highlight cell movement towards the anterior. Alternative link: https://youtu.be/60LWsAdNPGY. (MP4 3.02 mb) Additional file 9: Movie 9. Neoblast-like cells migrating along the nerve cord of a regenerating annelid worm (magnified view). Detail of Movie 8 showing a magnified view of the movement of neoblast-like slider cells on the dorsal surface of the nerve cord in a posteriorly regenerating annelid, *Pristina leidyi*. Green arrowheads highlight cell movement towards the posterior of the animal, while magenta arrowheads highlight cell movement towards the anterior. Alternative link: https://youtu.be/zOl8SZVD3sw. (MP4 802 kb) Additional file 10: Movie 10. Slider cells migrating during annelid head regeneration. High-spatial, fine-temporal resolution time-lapse recording of posterior regeneration after head amputation. The specimen was mounted using the slide-and-oil method and imaged using a Zeiss Axioscope 2 compound microscope. This clip shows anterior slider cell migration during anterior regeneration in the annelid worm *Pristina leidyi*. Notice the slider cell stopping to undergo cell division (mitosis) towards the end of the clip. Alternative link: https://youtu.be/KmCLaghVYaE. (MP4 1.02 mb) Additional file 11: Movie 11. Migrating cell undergoes mitosis during annelid head regeneration. Detail of Movie 10 showing an anterior slider cell stopping to undergo mitosis during anterior regeneration in the annelid worm *Pristina leidyi*. Alternative link: https://youtu.be/PgUi-S74zNM. (MP4 181 kb)

## Abbreviations

AP: antero-posterior
CRW: correlated random walk
CVM: correlated velocity
DV: dorso-ventral
hpa: hours post amputation
msd: mean square displacement
ν: mean tangential speed
SpW: artificial spring water
τ: time scale of autocorrelation
TTX: tetrodotoxin

## References

[1] Boot MJ, Westerberg CH, Sanz-Ezquerro J, Cotterell J, Schweitzer R, Torres M, Sharpe J. In vitro whole-organ imaging: 4D quantification of growing mouse limb buds. Nat Meth. 2008;5:609–12. 10.1038/nmeth.1219 18516047

[2] Öztürk G, Erdoǧan E (2004). Multidimensional long‐term time‐lapse microscopy of in vitro peripheral nerve regeneration. Microsc Res Tech.

[3] Walsh MK, Quigley HA (2008). In vivo time-lapse fluorescence imaging of individual retinal ganglion cells in mice. J Neurosci Methods.

[4] Yamagata K, Ueda J (2013). Long-term live-cell imaging of mammalian preimplantation development and derivation process of pluripotent stem cells from the embryos. Dev Growth Differ.

[5] Funahashi J, Nakamura H (2014). Time-lapse imaging system with shell-less culture chamber. Dev Growth Differ.

[6] Kulesa PM, Fraser SE (2000). In ovo time-lapse analysis of chick hindbrain neural crest cell migration shows cell interactions during migration to the branchial arches. Development.

[7] Kamei M, Weinstein BM (2005). Long-term time-lapse fluorescence imaging of developing zebrafish. Zebrafish.

[8] Kaufmann A, Mickoleit M, Weber M, Huisken J (2012). Multilayer mounting enables long-term imaging of zebrafish development in a light sheet microscope. Development.

[9] Schuster K, Ghysen A (2013). Time-lapse analysis of primordium migration during the development of the fish lateral line. Cold Spring Harb Protoc.

[10] Lisse TS, Brochu EA, Rieger S. Capturing tissue repair in zebrafish larvae with time-lapse Brightfield Stereomicroscopy. J Vis Exp. 2015;95:e52654 . 10.3791/52654 PMC4330669 25742070

[11] Du Z, Santella A, He F, Tiongson M, Bao Z (2014). De novo inference of systems-level mechanistic models of development from live-imaging-based phenotype analysis. Cell.

[12] Giurumescu CA, Kang S, Planchon TA, Betzig E, Bloomekatz J, Yelon D, Cosman P, Chisholm AD. Quantitative semi-automated analysis of morphogenesis with single-cell resolution in complex embryos. Development. 2012;139:4271–9. 10.1242/dev.086256 PMC3478691 23052905

[13] Schnabel R, Hutter H, Moerman D, Schnabel H (1997). Assessing normal embryogenesis in Caenorhabditis elegans using a 4D microscope: variability of development and regional specification. Dev Biol.

[14] Wheeler SR, Pearson JC, Crews ST (2012). Time-lapse imaging reveals stereotypical patterns of Drosophila midline glial migration. Dev Biol.

[15] Carroll DJ, Stricker SA, editors. Microinjection and 4D fluorescence imaging in the eggs and embryos of the Ascidian phallusia mammillata - Springer. Humana Press, New York. 2014 [Methods in Molecular Biology, vol. 1128]. 10.1007/978-1-62703-974-1_11 24567214

[16] Kurokawa H, Aoki Y, Nakamura S, Ebe Y, Kobayashi D, Tanaka M (2006). Time‐lapse analysis reveals different modes of primordial germ cell migration in the medaka Oryzias latipes. Dev Growth Differ.

[17] Strobl F, Stelzer EHK (2014). Non-invasive long-term fluorescence live imaging of Tribolium castaneum embryos. Development.

[18] Abdulreda MH, Faleo G, Molano RD, Lopez-Cabezas M, Molina J, Tan Y, Echeverria OAR, Zahr-Akrawi E, Rodriguez-Diaz R, Edlund PK, Leibiger I, Bayer AL, Perez V, Ricordi C, Caicedo A, Pileggi A, Berggren P-O. High-resolution, noninvasive longitudinal live imaging of immune responses. Proc Natl Acad Sci. 2011;108:12863–8. 10.1073/pnas.1105002108 PMC3150878 21768391

[19] Bestman JE, Lee-Osbourne J, Cline HT (2012). In vivo time-lapse imaging of cell proliferation and differentiation in the optic tectum of Xenopus laevis tadpoles. J Comp Neurol.

[20] Chai Y, Li W, Feng G, Yang Y, Wang X, Ou G (2012). Live imaging of cellular dynamics during Caenorhabditis elegans postembryonic development. Nat Protoc.

[21] Bradshaw B, Thompson K, Frank U (2015). Distinct mechanisms underlie oral vs aboral regeneration in the cnidarian Hydractinia echinata. eLife.

[22] Bely AE, Sikes JM (2010). Latent regeneration abilities persist following recent evolutionary loss in asexual annelids. Proc Natl Acad Sci.

[23] Elliott SA, Sánchez Alvarado A (2013). The history and enduring contributions of planarians to the study of animal regeneration. Wiley Interdiscip Rev Dev Biol.

[24] Monaghan J, Epp L, Putta S, Page R, Walker J, Beachy C, Zhu W, Pao G, Verma I, Hunter T, Bryant S, Gardiner D, Harkins T, Voss SR (2009). Microarray and cDNA sequence analysis of transcription during nerve-dependent limb regeneration. BMC Biol.

[25] Mookerjee S, Bhattacharjee A (1967). Regeneration time at the different levels of hydra. Wilhelm Roux Arch Für Entwicklungsmechanik Org.

[26] Poss KD, Keating MT, Nechiporuk A (2003). Tales of regeneration in zebrafish. Dev Dyn.

[27] Tanaka EM (2003). Cell differentiation and cell fate during urodele tail and limb regeneration. Curr Opin Genet Dev.

[28] Zattara EE, Bely AE (2011). Evolution of a novel developmental trajectory: fission is distinct from regeneration in the annelid Pristina leidyi. Evol Dev.

[29] Yates DM, Wolstenholme AJ (2004). An ivermectin-sensitive glutamate-gated chloride channel subunit from Dirofilaria immitis. Int J Parasitol.

[30] Budán F, Kovács N, Engelmann P, Horváth I, Veres DS, Németh P, Szigeti K, Máthé D. Longitudinal in vivo MR imaging of live earthworms. J Exp Zool Part Ecol Genet Physiol. 2014;321:479–89. 10.1002/jez.1880 25059556

[31] Narahashi T, Moore JW, Scott WR (1964). Tetrodotoxin blockage of sodium conductance increase in lobster giant axons. J Gen Physiol.

[32] Nakamura Y, Nakajima S, Grundfest H (1965). The action of tetrodotoxin on electrogenic components of squid giant axons. J Gen Physiol.

[33] Hidaka T, Ito Y, Kuriyama H (1969). Membrane properties of the somatic muscle (obliquely striated muscle) of the earthworm. J Exp Biol.

[34] Barna J, Csoknya M, Lázár Z, Barthó L, Hámori J, Elekes K (2001). Distribution and action of some putative neurotransmitters in the stomatogastric nervous system of the earthworm, Eisenia fetida (Oligochaeta, Annelida). J Neurocytol.

[35] Kumar A, Brockes JP (2012). Nerve dependence in tissue, organ, and appendage regeneration. Trends Neurosci.

[36] Simakov O, Marletaz F, Cho S-J, Edsinger-Gonzales E, Havlak P, Hellsten U, Kuo D-H, Larsson T, Lv J, Arendt D, Savage R, Osoegawa K, de Jong P, Grimwood J, Chapman JA, Shapiro H, Aerts A, Otillar RP, Terry AY, Boore JL, Grigoriev IV, Lindberg DR, Seaver EC, Weisblat DA, Putnam NH, Rokhsar DS (2013). Insights into bilaterian evolution from three spiralian genomes. Nature.

[37] Zattara EE, Bely AE (2013). Investment choices in post-embryonic development: quantifying interactions among growth, regeneration, and asexual reproduction in the annelid Pristina leidyi. J Exp Zoolog B Mol Dev Evol.

[38] Özpolat BD, Bely AE. Gonad establishment during asexual reproduction in the annelid Pristina leidyi. Dev Biol.2015;405(1):123-36. 10.1016/j.ydbio.2015.06.001 26134407

[39] Zattara EE. Regeneration, fission and the evolution of developmental novelty in naid annelids. Ph.D. Thesis. University of Maryland, College Park; 2012.

[40] Bilej M, Procházková P, Šilerová M, Josková R, Söderhäll K (2010). Earthworm immunity. Invertebrate immunity.

[41] Envall I, Erséus C, Gustavsson LM (2008). Ultrastructural investigation of coelomocytes in representatives of Naidinae and Rhyacodrilinae (Annelida, Clitellata, Tubificidae). J Morphol.

[42] Herlant-Meewis H (1964). Regeneration in annelids. Adv Morphog.

[43] Gurarie E, Ovaskainen O (2011). Characteristic spatial and temporal scales unify models of animal movement. Am Nat.

[44] Bely AE (2014). Early events in annelid regeneration: a cellular perspective. Integr Comp Biol.

[45] Randolph H (1891). The regeneration of the tail in Lumbriculus. Zool Anz.

[46] Randolph H (1892). The regeneration of the tail in Lumbriculus. J Morphol.

[47] Sugio M, Yoshida-Noro C, Ozawa K, Tochinai S. Stem cells in asexual reproduction of Enchytraeus japonensis (Oligochaeta, Annelid): proliferation and migration of neoblasts. Dev Growth Differ. 2012. 10.1111/j.1440-169X.2012.01328.x 22417296

[48] Bilello AA, Potswald HE (1974). A cytological and quantitative study of neoblasts in the naid Ophidonais serpentina (Oligochaeta). Wilhelm Roux Arch Entwicklungsmechanik Org.

[49] Foulkes RH (1953). Regeneration of the anterior end of Aulophorus furcatus (Naididae) with special reference to effect of x-rays. Biol Bull.

[50] Hill SD (1970). Origin of the regeneration blastema in polychaete annelids. Am Zool.

[51] Krecker FH (1923). Origin and activities of the neoblasts in the regeneration of microdrilous annelida. J Exp Zool.

[52] Myohara M (2012). What role do annelid neoblasts play? A comparison of the regeneration patterns in a neoblast-bearing and a neoblast-lacking Enchytraeid oligochaete. PLoS One.

[53] O’Brien JP (1946). Studies on the cellular basis of regeneration in Nais paraguayensis, and the effects of x-rays thereon. Growth.

[54] Stone RG (1932). The effects of x-rays on regeneration in Tubifex tubifex. J Morphol.

[55] Stone RG (1933). The effects of X-rays on anterior regeneration in Tubifex tubifex. J Morphol.

[56] Tadokoro R, Sugio M, Kutsuna J, Tochinai S, Takahashi Y (2006). Early segregation of germ and somatic lineages during gonadal regeneration in the annelid Enchytraeus japonensis. Curr Biol.

[57] Turner CD (1934). The effects of x-rays on posterior regeneration in Lumbriculus inconstans. J Exp Zool.

[58] Turner CD (1935). The effects of x-rays on anterior regeneration in Lumbriculus inconstans. J Exp Zool.

[59] Sayles LP (1927). Origin of the mesoderm and behaviour of the nucleolus in regeneration in Lumbriculus. Biol Bull.

[60] Krecker FH (1910). Some phenomena of regeneration in Limnodrilus and related forms. Z Wissenschlaftliche Zool.

[61] Zattara EE, Bely AE (2015). Fine taxonomic sampling of nervous systems within Naididae (Annelida: Clitellata) reveals evolutionary lability and revised homologies of annelid neural components. Front Zool.

[62] Abramoff MD, Magalhaes PJ, Ram SJ (2004). Image processing with ImageJ. Biophotonics Int.

[63] Johnson DS, London JM, Lea M-A, Durban JW (2008). Continuous-time correlated random walk model for animal telemetry data. Ecology.

[64] R Development Core Team (2011). R: a language and environment for statistical computing. Vienna, Austria.

[65] Zattara EE, Turlington KW, Bely AE. Long-term time-lapse live imaging reveals extensive cell migration during annelid regeneration. Zenodo. 2016. 10.1186/s12861-016-0104-2 PMC4804569 27006129

